# Expression of the MOZ-TIF2 oncoprotein in mice represses senescence

**DOI:** 10.1016/j.exphem.2015.12.006

**Published:** 2016-04

**Authors:** Anne Largeot, Flor Maria Perez-Campo, Elli Marinopoulou, Michael Lie-a-Ling, Valerie Kouskoff, Georges Lacaud

**Affiliations:** a Cancer Research UK Stem Cell Biology Group, CR-UK Manchester Institute, University of Manchester, Manchester, UK; b Cancer Research UK Stem Cell Hematopoiesis Group, CR-UK Manchester Institute, University of Manchester, Manchester, UK

## Abstract

The MOZ-TIF2 translocation, which fuses monocytic leukemia zinc finger protein (MOZ) histone acetyltransferase (HAT) with the nuclear co-activator TIF2, is associated with the development of acute myeloid leukemia. We recently found that in the absence of MOZ HAT activity, *p16*^*INK4a*^ transcriptional levels are significantly increased, triggering an early entrance into replicative senescence. Because oncogenic fusion proteins must bypass cellular safeguard mechanisms, such as senescence and apoptosis, to induce leukemia, we hypothesized that this repressive activity of MOZ over *p16*^*INK4a*^ transcription could be preserved, or even reinforced, in MOZ leukemogenic fusion proteins, such as MOZ-TIF2. We describe here that, indeed, MOZ-TIF2 silences expression of the *CDKN2A* locus (*p16*^*INK4a*^ and *p19*^*ARF*^), inhibits the triggering of senescence and enhances proliferation, providing conditions favorable to the development of leukemia. Furthermore, we describe that abolishing the MOZ HAT activity of the fusion protein leads to a significant increase in expression of the *CDKN2A* locus and the number of hematopoietic progenitors undergoing senescence. Finally, we report that inhibition of senescence by MOZ-TIF2 is associated with increased apoptosis, suggesting a role for the fusion protein in p53 apoptosis-versus-senescence balance. Our results underscore the importance of the HAT activity of MOZ, preserved in the fusion protein, for repression of the *CDKN2A* locus transcription and the subsequent block of senescence, a necessary step for the survival of leukemic cells.

## Highlights

• MOZ-TIF2 transformation is associated with inhibition of senescence through repression of the *CDKN2A* locus.
• MOZ histone acetyltransferase activity of MOZ-TIF2 is required for the inhibition of senescence.
• MOZ-TIF2 acts on the p53 apoptosis-versus-senescence balance.

The monocytic leukemia zinc finger protein (MOZ, MYST3, KAT6A) is the founding member of the MYST family of histone acetyltransferases (HATs) 1, 2, 3. MOZ is essential for hematopoietic stem cell (HSC) emergence and self-renewal 4, 5, 6. The gene encoding MOZ was initially identified in several recurrent chromosomal translocations, with either CBP, p300, or TIF2/NCOA2 found in a distinct subtype of acute myeloid leukemia (AML) with French–American–British M4/5 morphology characterized by a poor prognosis 2, 7, 8, 9, 10. Remarkably, the HAT domain of MOZ is preserved in the fusion protein, and all the fusion partners of MOZ are themselves directly (CBP, P300) or indirectly (TIF2 can interact with CBP via the CBP interaction domain or CID [11]) involved in posttranslational histone modifications and transcriptional regulation. This observation led to the proposition that abnormal histone acetylation driven by the fusion proteins might be at the origin of the leukemic transformation 12, 13. Alternatively, it has been proposed that the ability of MOZ-TIF2 to deplete CBP, particularly from the promyelocytic leukemia (PML) bodies, results in subversion of normal gene expression leading to development of leukemia 14, 15, 16.

MOZ-TIF2 is able to immortalize murine hematopoietic progenitors in vitro and to recapitulate AML in vivo in murine and zebrafish models 13, 17, 18, 19. Previous reports have indicated that a functional CID or MOZ HAT activity is required to increase the proliferative potential of hematopoietic progenitors in vitro, and to induce AML in vivo 13, 17, 20.

Cells acquiring oncogenic mutations or translocations need to evade defense mechanisms, such as senescence and apoptosis, to survive and proliferate. In this context, MOZ has been found to regulate, upon cellular stress, expression of the tumor suppressor gene p21 and to increase premature senescence through acetylation of P53 21, 22. In contrast to this positive role of MOZ in inducing senescence, we, and others, have reported that in the absence of MOZ, mouse embryonic fibroblasts (MEFs) undergo an early entrance into replicative senescence mediated by the upregulation of expression from the *CDKN2A* locus (*p16*^*INK4a*^ and *p19*^*ARF*^) 23, 24. These observations raise the possibility that this repressive activity could be exacerbated in MOZ leukemic fusion proteins. In this work, we sought to investigate this possibility by determining the effect of MOZ-TIF2 expression on the transcriptional levels of *p16*^*INK4a*^/*p19*^*AR*F^ and proliferation of the targeted cells. We also investigated the relevance of the HAT activity of MOZ, preserved in all known leukemic proteins originated by MOZ translocation, in this context.

## Methods

### Purification of cKit^+^ cells

Bone marrow cells were stained with a biotinylated anti-cKit antibody (BD Biosciences, clone 2B8m 553353) and incubated with the anti-biotin magnetic MACS beads (Miltenyi Biotec). cKit^+^ cells were enriched using an LS column and a MACS Separator magnetic isolation device (Miltenyi Biotec).

### Flow cytometry

Embryoid bodies (EBs), generated as previously described [25], or MEFs were trypsinized (TryplE, Gibco). Stained single-cell suspensions were analyzed on a FACScan or a FACS Calibur flow cytometer (Becton Dickinson) or sorted on a FACS Vantage cell sorter (Becton Dickinson). Cell cycle analysis was performed using the Click-iT EdU Alexa Fluor 647 Flow Cytometry Assay Kit (Thermo Fisher Scientific). Apoptosis analysis was performed using the PE Annexin V Apoptosis Detection Kit (BD Biosciences).

### Senescence-associated β−galactosidase staining

Senescence-associated β-galactosidase (SA-β-Gal) activity was detected using the Senescence β-galactosidase Staining Kit from Cell Signalling.

### Chromatin immunoprecipitation assay

Chromatin immunoprecipitation (ChIP) was performed using the High Cells Chip Kit (Diagenode) following the instructions of the manufacturer.

Additional information concerning other techniques and materials can be found in the Supplementary Methods (online only, available at www.exphem.org) 26, 27, 28, 29.

## Results and discussion

**Supplementary Figure E1:**
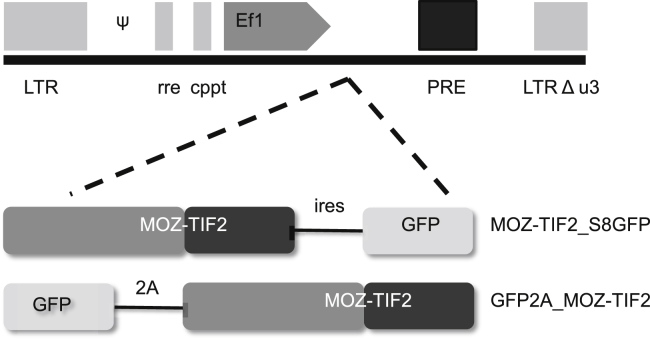
Schematic overview of the MOZ-TIF2-expressing lentiviral vectors used in this study. Ef1 = human elongation factor 1α promoter; LTR = long terminal repeat; ψ = viral packaging signal; rre = rev responsive elements; cppt = central polypurine tract; PRE = hepatitis B virus posttranscriptional regulatory element; GFP = enhanced green fluorescent protein; ires = internal ribosomal entry site, 8 repeats of the 9-nt IRES module from the Gtx 5′ UTR; 2a = foot-and-mouth disease virus self-cleaving 2a peptide sequence -F2A.

**Figure 1:**
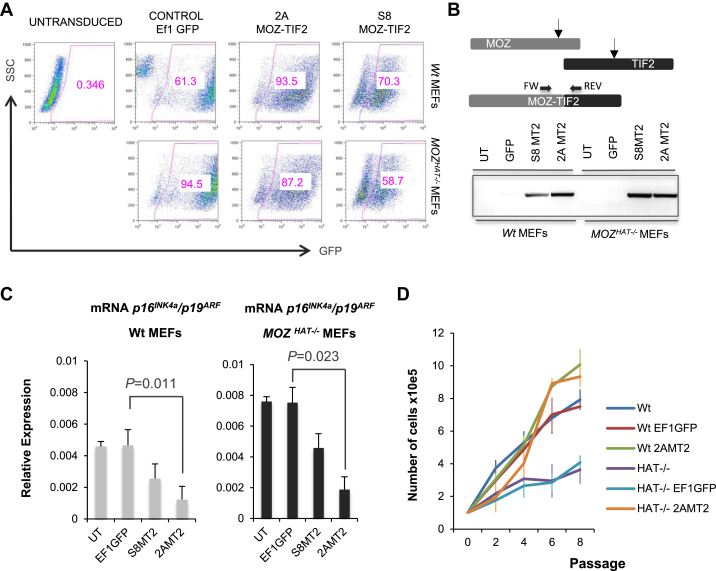

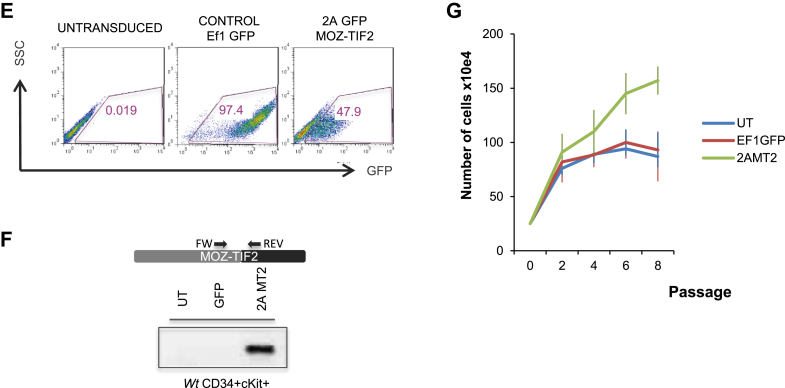
(**A**) Flow cytometry profile of untransduced MEFs (UT) and MEFs transduced with the different lentiviruses (EF1GFP, 2AGFP_MOZTIF2, and S8MOZ-TIF2) and therefore expressing GFP. Numbers represent the percentages of cells positive for GFP 48 hours after transduction in each case (*n* = 3 for each genotype). Multiplicity of infection (MOI) = 30. (**B**) PCR for the MOZ-TIF2 transcript on untransduced cells and MEFs transduced with the different lentiviruses. (**C**) Quantitative PCR revealing the relative expression levels of *p16*^*INK4a*^/*p19*^*ARF*^ in WT and *Moz*^HAT–/–^ MEFs (passage 3) untransduced or transduced with the different lentiviruses. The transcript levels were normalized to β-actin for all reactions. Values reflect averages of triplicate samples. Bars represent standard errors of the mean values. (**D**) Growth curves of cultures of WT and *Moz*^HAT–/–^ MEFs transduced with the different lentiviruses. The graph represents the average values from three independent cultures. Passage numbers are indicated. Bars represent standard errors of the mean values. (**E**) Flow cytometry profile of untransduced (UT) CD34^+^cKit^+^ hematopoietic progenitors and the same cells transduced with either EF1GFP or 2AGFP_MOZTIF2 lentivirus. Numbers represent the percentages of cells positive for GFP 48 hours after transduction in each case. MOI = 50. (**F**) Specific PCR for the detection of MOZ-TIF2 transcripts in transduced CD34^+^cKit^+^ cells. (**G**) Growth curves of WT CD34^+^cKit^+^ cultures transduced with the different lentiviruses. The graph represents the average values from three independent cultures. Passage numbers are indicated. Bars indicate standard errors of the mean values.

To assess the effect of MOZ-TIF2 oncoprotein on the transcriptional levels of the *CDKN2A* locus (*p16*^*INK4a*^*/p19*^*ARF*^*)*, we first transduced either wild-type (WT) or *MOZ*^*HAT–/–*^ MEFs with two vectors linking MOZ-TIF2 to GFP either through a small S8 IRES or through a self-cleaving 2A peptide sequence (Supplementary Figure E1, online only, available at www.exphem.org). Untransduced cells, as well as cells transduced with a lentivirus expressing only GFP (EF1GFP), were used as controls (Fig. 1A). Polymerase chain reactions (PCRs) confirmed the presence of MOZ-TIF2 transcripts in the transduced cells (Fig. 1B). A significant reduction in *p16*^*INK4a*^/*p19*^*ARF*^ mRNA levels was observed not only in WT MEFs, but also in *MOZ*^*HAT–/–*^ MEFs, which, as previously reported [23], express higher levels of *p16*^*INK4a*^/*p19*^*ARF*^ than the WT cells (Fig. 1C). As the GFP-2A-MOZ-TIF2 lentivirus (2AMT2) had higher transduction efficiency (Fig. 1A), we chose this virus to perform the subsequent experiments.

We next analyzed whether the lower levels of the *CDKN2A* locus transcription were correlated with changes in proliferation upon successive passages. As expected, the *MOZ*^*HAT–/–*^ MEFs proliferated less than the WT MEFs. This difference was maintained following transduction with EF1GFP viruses (Fig. 1D). In contrast, expression of MOZ-TIF2 conferred a clear growth advantage to both WT and *MOZ*^*HAT–/–*^ MEFs (Fig. 1D). These results suggest that overexpression of the MOZ-TIF2 fusion protein is able to counteract the effect of the native MOZ protein and its HAT deficient version.

To test whether the effect of MOZ-TIF2 expression was also observed in hematopoietic progenitors, we transduced WT CD34^+^cKit^+^ cells isolated from day 6 in vitro differentiated embryonic stem cells with the 2AMT2 lentivirus (Fig. 1E). PCRs on sorted GFP^+^ cells confirmed the presence of MOZ-TIF2 transcripts in these cells (Fig. 1F). Similarly to MEFs, CD34^+^cKit^+^ hematopoietic progenitors transduced with MOZ-TIF2 had an increased proliferation rate compared with the control cells (Fig. 1G).

**Figure 2:**
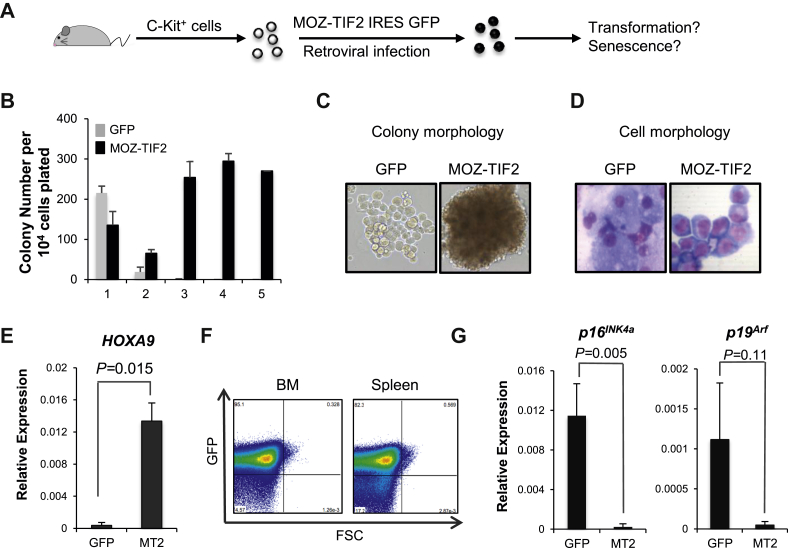

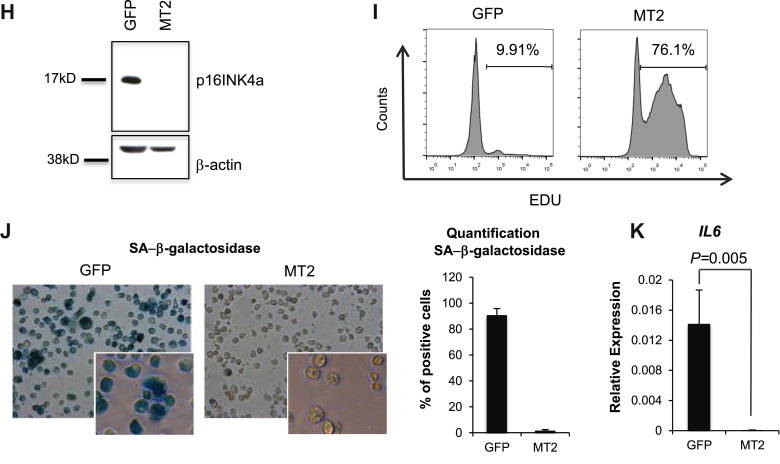
(**A**) Schematic representation of the experimental design. Bone marrow cKit^+^ cells infected with a retrovirus encoding MOZ-TIF2 were tested for their leukemic potential and senescence status. (**B**) Serial replating of MOZ-TIF2 (MT2)-expressing cells and GFP control cells (*n* = 3). (**C**) Photographs of the colonies after the second replating. Representative images from three independent experiments. (**D**) MGG staining of cytospin from the second replating. Representative images from three independent experiments. (**E**) Analysis of *HOXA9* transcript levels in cKit^+^ expressing MOZ-TIF2 or the control virus (*n* = 3). (**F**) Flow cytometry detection of MOZ-TIF2-expressing cells (GFP^+^) in the bone marrow (BM) and the spleen of mice culled because of sickness. Representative FACS plots of five mice. (**G**) Analysis of *p16*^*INK4a*^/*p19*^*ARF*^ transcript levels in cKit^+^ expressing MOZ-TIF2 or cells transduced with the control virus (*n* = 3). (**H**) Western blot analysis for p16^INK4a^ protein levels in the MOZ-TIF2 and control cells. Results are representative of two independent experiments. (**I**) Flow cytometry analysis of cell cycle. (**J**) Photographs of MT2 and control cells after SA-β-Gal staining and quantification (*n* = 3). (**K**) Analysis of *IL6* transcript levels in cKit^+^ expressing MOZ-TIF2 or in control cells (*n* = 3).

We then studied the effect of MOZ-TIF2 on senescence in leukemic cells transformed with this fusion protein. cKit^+^ cells isolated from adult WT mouse bone marrow were transduced with MOZ-TIF2 and control retroviruses (Fig. 2A). As previously described 17, 20, cells transduced with MOZ-TIF2, but not with the GFP control viruses, could be serially replated in vitro in methylcellulose cultures (Fig. 2B), presented a blast morphology (Fig. 2C, D), and expressed high levels of the homeotic gene *HoxA9* (Fig. 2E). Furthermore, these cells, when injected into sublethally irradiated recipients, induced the development of fully penetrant leukemia with concomitant invasion of hematopoietic organs with GFP^+^ (MOZ-TIF2-expressing) cells (Fig. 2F). This leukemic transformation induced by MOZ-TIF2 was associated with a decrease in expression of both genes encoded in the CDKN2A locus, *p16*^*INK4a*^ and p*19*^*ARF*^ (Fig. 2G), a decrease in p16^INK4a^ protein level (Fig. 2H), and a marked increase in cell division (Fig. 2I). Accordingly, only a low percentage of cells expressing the MOZ-TIF2 fusion protein were positive for SA-β-Gal (Fig. 2J), in contrast to control GFP^+^ cells. In agreement with these results, cells transduced with the MOZ-TIF2 fusion protein also expressed much lower levels of interleukin (IL)-6, a member of the senescence-associated secretory pathway (Fig. 2K), compared with the cells transduced with the control vector. Together, these results clearly indicate that expression of the MOZ-TIF2 fusion protein, though inducing the development of leukemia, inhibits the triggering of senescence.

**Figure 3:**
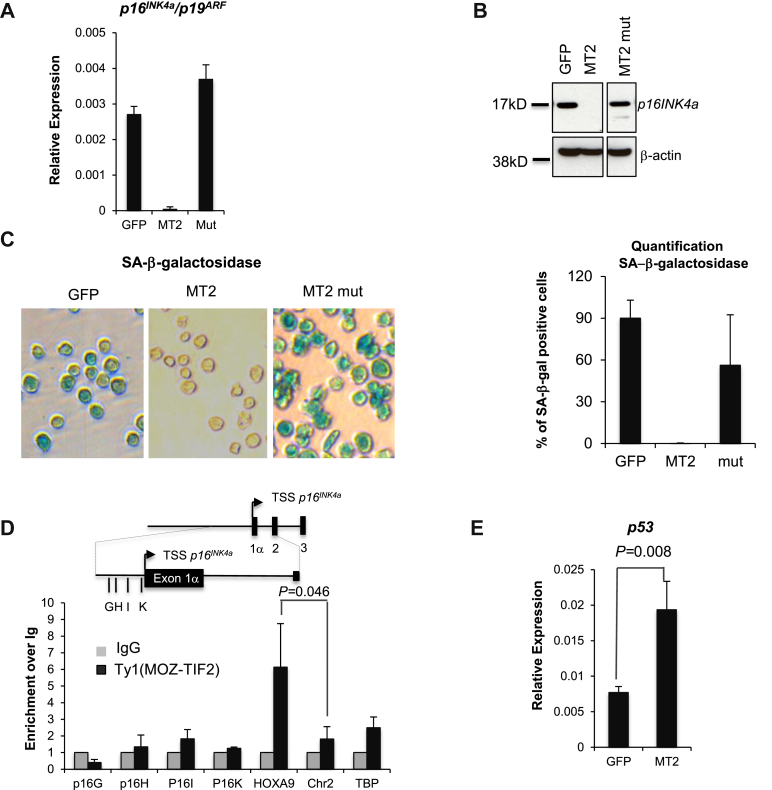

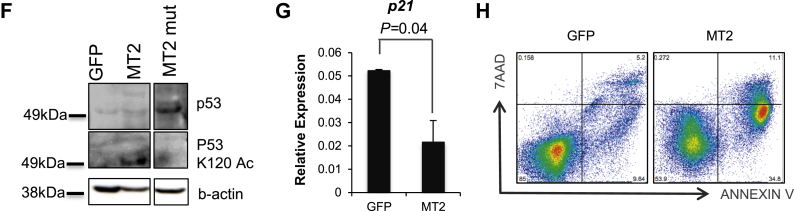
(**A**) Analysis of *p16*^*INK4a*^/*p19*^*ARF*^ transcript levels in cKit^+^ cells expressing MOZ-TIF2, MOZ-TIF2 with the mutated HAT domain of MOZ, or the control virus (*n* = 2). (**B**) Western blot analysis of p16^INK4a^ protein levels in the cells expressing the mutated form of MOZ-TIF2 compared with WT MOZ-TIF2 cells. (**C**) Photographs of cells expressing either the WT or the mutant MOZ-TIF2 fusion protein and control cells after SA-β-Gal staining (*left*) and quantification (*right*) (*n* = 3). (**D**) ChIP analysis of the recruitment of the Ty1-tagged MOZ-TIF2 using an anti-Ty1 antibody (*n* = 2). (**E**) Analysis of *p53* transcript levels in cKit^+^ expressing MOZ-TIF2 or the control virus (*n* = 3). (**F**) Western blot analysis of p53 and p53 acetylated at lysine 120 (p53K120) protein levels in cells expressing MOZ-TIF2 or control cells. (**G**) Analysis of *p21* transcript levels in cKit^+^ expressing MOZ-TIF2 or the control virus (*n* = 3). (**H**) Flow cytometry analysis of apoptosis using annexin V and 7ADD staining. Results are representative images from two independent experiments.

To determine the relevance of MOZ HAT activity, we then transduced cells with a mutated version of MOZ-TIF2 (Q654E/G657E) that abrogates this activity [20]. We detected dramatically higher expression levels of *p16*^*Ink4a*^/p19^ARF^ mRNA, p16^Ink4^ protein, and SA-β-Gal in these cells than in cells transduced with MOZ-TIF2-expressing viruses (Fig. 3A–C). These results suggest that the HAT activity of MOZ is crucial to avoid replicative senescence.

We next wanted to check if the repression of *p16*^*INKa*^ was a direct effect of the binding of the MOZ-TIF2 fusion protein to the *p16*^*INKa*^ promoter. We therefore performed chromatin immunoprecipitation (ChIP) analysis using a Ty1-tagged MOZ-TIF2. Although we detected good enrichment of the fusion protein at the *HOXA9* promoter (a known target of MOZ or MOZ-TIF2) 20, 30, compared with the two negative controls (condensed region in chromosome 2 or promoter of the *TBP* gene), we did not detect clear enrichment of MOZ-TIF2 recruitment into the *p16*^*Ink4a*^ promoter (Fig. 3D). These data suggest that MOZ-TIF2 is repressing *p16*^*INKa*^ expression in an indirect manner.

In addition to its role in inhibition of senescence through the *CDKN2A* locus, MOZ has been reported to acetylate p53 to activate p21-dependent senescence after DNA damage 21, 22. We therefore investigated the status of this pathway in MOZ-TIF2 leukemic cells and detected an increase in the transcriptional levels of *p53* (Fig. 3E) in these cells. Furthermore expression of MOZ-TIF2 was associated with K120 acetylation of p53, whereas no acetylated p53 was detected in cells expressing the control vector or the mutated MOZ-TIF2 (Fig. 3F). Surprisingly, *p21* transcription levels were however reduced, and not increased, upon expression of the MOZ-TIF2 fusion protein (Fig. 3G). Although this is consistent with the decrease in the number of cells undergoing replicative senescence, it does not reflect the previously described positive effect of K120 acetylation of p53 on *p21* expression 21, 22, 31, 32. Furthermore we detected a striking increase in cells undergoing apoptosis in MOZ-TIF2 leukemic cells compared to cells transduced with the control virus (Fig. 3H). Together our results suggest that MOZ-TIF2 alters the p53 apoptosis-versus-senescence balance in favor of apoptosis. The exact molecular regulation of this balance, and in particular the role of acetylation of p53 on lysine K120 in this process, is not yet fully understood. Indeed, although p53 K120 acetylation and p53-dependent p21 transcription are prevented by deletion of MOZ or TIP60, suggesting a direct correlation between these events 21, 22, 33, mutation of K120 of p53 results in a decrease in the capacity of p53 to activate apoptosis, but has, in contrast, no effect on *p21* transcription 31, 32.

We conclude from this work that the expression of MOZ-TIF2 fusion protein represses the transcription of *p16*^*INK4a*^ and *p19*^*ARF*^ and blocks senescence, and that the MOZ HAT activity of the fusion protein is crucial for this repressive activity. We propose that silencing of the *CDKN2A* locus by MOZ fusion proteins could be an important step in the expansion of cells harboring these oncogenic mutations. Moreover, MOZ-TIF2 seems to be acting on p53 apoptosis-versus-senescence balance. Finally, as *p16*^*INK4a*^ expression is a frequent target gene inactivated in human cancers, our work also raises the prospect that targeting the epigenetic HAT activity of MOZ [34] could represent an interesting strategy to induce senescence and eliminate oncogenic cells.

## Figures and Tables

**Supplementary Table E1:** Primers for qPCR

	Universal probes from Roche	Sequence
mouse p16 + p19	81	CGACGGGCATAGCTTCAG
GCTCTGCTCTTGGGATTGG
mouse p21	16	AACATCTCAGGGCCGAAA
TGCGCTTGGAGTGATAGAAA
mouse p53	4	GCAACTATGGCTTCCACCTG
TTATTGAGGGGAGGAGAGTACG
mouse p19	106	GGGTTTTCTTGGTGAAGTTCG
TTGCCCATCATCATCACCT
mouse p16	SYBR	GTACCCCGATTCAGGTGATG
CGAATCTGCACCGTAGTTGA
mouse IL6	6	GCTACCAAACTGGATATAATCAGGA
CCAGGTAGCTATGGTACTCCAGAA
mouse beta actin	106	TGACAGGATGCAGAAGAAGA
CGCTCAGGAGGAGCAATG

Primers for ChIP		

mouse HOXA9 promoter	SYBR	GAGCGGTTCAGGTTTAATGC
TGCCTGCTGCAGTGTATCAT
mouse p16G promoter	SYBR	ACTCAGCTTGCTTGGTAGCAG
GTTGGCCCTGCTTCTGTC
mouse p16H promoter	SYBR	AATGCCAGGCCTTTAATCCT
CCTGGAACTCAGCATAAACTCA
mouse p16I promoter	SYBR	TTCTAATACCTGGGTGTTGCAC
AAAGTGAACTAGTCCTTCTCGAAATC
mouse p16k promoter	SYBR	TCTGGAGCAGCATGGAGTC
GGGGTACGACCGAAAGAGTT
Chr2	SYBR	AGGGATGCCCATGCAGTCT
CCTGTCATCAGTCCATTCTCCAT
mouse TBP promoter	SYBR	CCGCAGTGCCCAGGTAACGG
GGGACCCGCTGCAGAAGTCG

**Supplementary Table E2:** Antibodies

	Application	Company	Clone/catalog no.	Dilution/quantity
p16^INK4a^	Western blot	Sigma	SAB4500072	1/2,000
β-Actin	Western blot	Sigma	A1978	1/10,000
p53	Western blot	Abcam	ab78316	1/1,000
p53 k120 Ac	Western blot	Abcam	ab26	1/1,000
Ty1	ChIP	Diagenode	C15200054	6 μg per IP
IgG isotype	ChIP	Diagenode	kch-803-015	6 μg per IP
c kit biotinylated	Purification of ckit^+^ cells	BD Biosciences	Clone 2B8m, 553353	1.5 μL for 10 × 10^6^ cells

## Supplementary methods

### Primers for quantitative polymerase chain reaction

See Supplementary Table E1.

### Lentiviral and retroviral constructs

The MOZ-TIF2 cDNA and the retroviral MSCV-MOZ-TIF2-IRES-GFP construct were kind gifts from Dr. Brian Huntly. To perform chromatin immunoprecipitation (ChIP), a 3XTy1 tag was added in frame at the 5′ extremity of the MOZ-TIF2 coding sequence. The mutant was generated using the Quickchange II XL Site-Directed Mutagenesis Kit (Agilent Technologies) by two steps of mutagenesis. The sequences of the primers used for mutagenesis are:

From WT to G657E:

5′-cctcaataccagcgtaaggaatatggcaggtttctcatcg
5′-cgatgagaaacctgccatattccttacgctggtattgagg

From G657E to Q654E/G657E:

5′- ctgccatattccttacgctcgtattgaggaagaatcatta
5′-taatgattcttcctcaatacgagcgtaaggaatatggcag

All third-generation lentiviral transfer vectors used in this study have the same backbone as EF1-eGFP [26] and were constructed by replacing the eGFP gene with the expression cassette of interest. The MOZ-TIF2 cDNA was cloned into PCR2.1 TOPO (Invitrogen), creating TOPO-MOZ-TIF2. For the bicistronic MOZ-TIF2_S8GFP lentiviral vector, the MOZ-TIF2 cDNA was cloned upstream of 8 repeats of the 9-nt IRES module from the Gtx 5′-UTR followed by eGFP [27]. The GFP2a_MOZ-TIF2 lentiviral vector was constructed by fusing eGFP to the foot-and-mouth disease virus self-cleaving 2a peptide sequence (F2A) [28] and the first 256 bp of the MOZ-TIF2 cDNA by PCR. The resulting fragment was sequenced and used to replace the first 256 bp of MOZ-TIF2 in TOPO-MOZ-TIF2, creating TOPO-GFP2a-MOZ-TIF2. Subsequently the GFP2a-MOZ-TIF2 cassette was cloned into the lentiviral backbone. Supplementary Figure E1 is a schematic of the MOZ-TIF2 vectors.

### Antibodies

See Supplementary Table E2.

### Lentiviral and retroviral constructs, virus production and infection of the cells

The VSVg pseudotyped lentiviral particles were produced using a third-generation self-inactivating lentiviral vector system. Briefly, viral supernatant was harvested from transfected HEK293T cells 2, 3, and 4 days posttransfection. Supernatant was passed through a 0.45-μm filter (Millipore), concentrated by ultracentrifugation (2 hours at 20,000 rpm, 47,000K, 4°C; Optima L-90K ultracentrifuge; Beckman Coulter) and resuspended in phosphate-buffered saline. Viral titres were expressed as HeLa cell transducing units per milliliter (TU/mL) as determined by flow cytometry for eGFP (FACS Calibur; Becton-Dickinson Biosciences) 72-h posttransduction.

Retroviral particles were produced using the Plat-E packaging cell line as described previously [29]. Briefly, viral supernatant was harvested from transfected Plat-E cells 2 and 3 days posttransfection. Supernatant was passed through a 0.45-μm filter (Millipore).

### Infection of c Kit+ cells with the retrovirus

Bone marrow from adult (10–14 weeks) WT mice was flushed from the femurs. After red blood cell lysis, the cells were stained with a biotinylated anti c-Kit antibody (BD Biosciences, Clone 2B8m 553353) and incubated with anti-biotin magnetic MACS beads (2 μL for 10^6^ cells, Miltenyi Biotec). After being washed with 10% serum in phosphate-buffered saline, the c Kit^+^ cells were enriched using an LS column and the manual MACS Separator magnetic isolation device (Miltenyi Biotec) accordingly to the manufacturer's protocol. The c Kit^+^ cells were infected using retroviral supernatants and retronectin, following the manufacturer's protocol (Takara, Clontech). Briefly, 10 μg of retronectin was coated in a noncoated 48-well plate for 2 hours at room temperature, the plate was then blocked for 30 min with 1% phosphate-buffered saline–bovine serum albumin, and the retroviral supernatant was added to the well and centrifuged for 2 hours at 1,500*g* at 32°C. After removal of the supernatant, 50,000 cells were added, and cells were incubated from 24 to 48 hours.

### Expression analysis by PCR and qPCR

RNAs were extracted with the RNeasy kit from Qiagen. Half a microgram of RNA was subjected to reverse transcription using the high-capacity cDNA reverse transcription kit (Thermofisher Scientific). The Universal PCR Master Mix or the Power SYBR Green Master Mix (Thermofisher Scientific) was used for the quantitative PCRs.

### Serial replating assay

Forty-eight hours after infection, 10,000 cells infected with the retrovirus coding for MOZ-TIF2 IRES GFP or for GFP only were plated in a semisolid methylcellulose-based medium containing stem cell factor, interleukin-3, granulocyte–macrophage colony-stimulating factor, TPO (obtained from the supernatant of producing cell lines, 1/100 dilution), interleukin-11, erythropoietin, interleukin-6, and mouse colony-stimulating factor (R&D System), in a 35-mm dish (Corning). After 7 days in culture, the colonies were counted. The cells were then harvested and replated in a new dish.

### Cytospin and MGG staining

Fifty thousand cells were cytospun at 800 rpm for 5 min on a microscopy slide. The cells were then stained for 3 min in the May–Grunwald staining solution (VWR Chemicals), rinsed in water, and stained for 20 min in the Giemsa staining solution diluted 20 times in water (VWR Chemicals).

### In vivo assay of leukemic potential of MOZ-TIF2-expressing c Kit^+^ cells

Twenty-four hours after infection, 500,000 c Kit^+^ cells from PEP3 mice (CD45.1) infected with the MSCV-MOZ-TIF2-IRES-GFP retrovirus were injected into irradiated C57/Bl6 recipient mice (CD45.2). All animal work was performed under regulation in accordance with the UK Animal Scientific Procedures Act (ASPA) 1986. Animal experiments were approved by the Animal Welfare and Ethics Review Body (AWERB) of the Cancer Research UK Manchester Institute.

### Flow cytometry

Cell cycle analysis was performed using the Click-iT EdU Alexa Fluor 647 Flow Cytometry Assay Kit following the manufacturer's protocol (Thermo fisher scientific). After 30 min of incubation with EdU, the cells were fixed and permeabilized. Detection of EdU was performed with the Click-It dye conjugated with AF647, and DNA content was stained with FxViolet. Apoptosis analysis was performed using the PE Annexin V Apoptosis Detection Kit (BD Biosciences). The cells were washed and stained with 5 μL of PE Annexin V and 7 μL of 7-AAD for 15 min at room temperature in the dark.

### Western blot

Thirty micrograms of proteins were loaded in a precast gel (Nupage, Invitrogen), and subjected to migration for 1 hour 30 min at 100 V. The proteins were transferred onto a nitrocellulose membrane using the iBlot device; the blocking and antibody probing were performed using the iBind device (Invitrogen). The antibodies used are listed in Supplementary Table E2.

### Chromatin immunoprecipitation assay

Chromatin immunoprecipitation was performed using the High Cells Chip Kit (Diagenode) following the instructions of the manufacturer. Five million cells were fixed for 8 min with 1% formaldehyde; after being washed with phosphate-buffered saline, the nucleus were isolated and sonicated for 15 cycles (30 sec on/30 sec off) with the Bioruptor NGS (Diagenode). The antibodies and quantities used for immunoprecipitations are listed in Supplementary Table E2. The pull-down experiments were conducted overnight. Eluted chromatin was quantified by quantitative PCR. Data were expressed as enrichment over Ig control.

### Statistics

The error bars in the graphs represent standard deviations. Statistical comparisons of data sets were performed with the two-tailed Student's *t* test.
